# An Overview of Dentist–Patient Communication in Quality Dental Care

**DOI:** 10.3390/dj13010031

**Published:** 2025-01-14

**Authors:** Jasmine Cheuk Ying Ho, Hollis Haotian Chai, Bella Weijia Luo, Edward Chin Man Lo, Michelle Zeping Huang, Chun Hung Chu

**Affiliations:** 1 Faculty of Dentistry, The University of Hong Kong, Hong Kong SAR 999077, China; jas_ho1218@connect.hku.hk (J.C.Y.H.); htchai89@connect.hku.hk (H.H.C.); bellaluo@hku.hk (B.W.L.); hrdplcm@hku.hk (E.C.M.L.); 2 Department of English, The Hang Seng University of Hong Kong, Hong Kong SAR 999077, China; michellehuang@hsu.edu.hk

**Keywords:** dentist–patient communication, medical communication, dentist–patient interaction

## Abstract

Dentist–patient communication is at the core of providing quality dental care. This study aims to review the importance, challenges, strategies, and training of dentist–patient communication. The World Dental Federation (FDI) emphasizes the importance of effective communication between oral healthcare providers and patients as a critical component of high-quality care. Effective dentist–patient communication allows dentists to accurately and effectively pass on essential medical information to patients. It improves the dentist’s efficiency, boosts self-confidence, reduces occupational stress, and minimizes the risks of complaint or litigation. Moreover, it alleviates dental anxiety and fear, helps build trust between dentists and patients, addresses patients’ needs and preferences, increases patients’ adherence to improved treatment outcomes, and ultimately leads to enhanced patient satisfaction. Nonetheless, it has been widely acknowledged that dentists universally encounter the repercussions arising from suboptimal communication strategies. Time constraints, difficulties in establishing rapport, the oral-health illiteracy of the patients, the poor communication skills of the dentists, dentists’ perceptions, and language barriers often hinder dentist–patient communication. Dentists should take the patient-centered approach as a premise and acquire verbal and non-verbal communication skills to overcome these communication barriers. The patient-centered approach comprises the understanding of patients’ illness, shared decision-making, and intervention with mindfulness of the patient’s own pace. Simple, succinct, and jargon-free language should be used in verbal communication. Proper body postures and gestures are fundamental for showing positive attitudes towards patients. Communication training for dental students should involve a structured pedagogical approach that includes didactic instruction, role-playing exercises, patient interviewing, and ongoing assessments. Key components of effective communication skills training in dental education include motivational interviewing, open-ended questioning, affirmations, reflective listening, and summaries to enhance patient engagement and adherence to treatment plans.

## 1. Introduction

The World Dental Federation (FDI) places significant emphasis on the importance of dentist–patient interaction in the *FDI Vision 2030: Delivering optimal oral health for all* [1]. The FDI advocates for patient-centered care, a healthcare approach that values and respects the preferences and needs of patients. At the heart of patient-centered care is effective communication. In the realm of medicine, there is a shared understanding of the essential elements of effective physician–patient communication during medical encounters, which include: (1) establishing rapport, (2) acquiring information, (3) sharing information, (4) involving patients in decision-making, (5) addressing emotional needs, and (6) supporting disease- and treatment-related behaviors [2]. The World Dental Federation defines quality in dental care as a collaborative process where dental professionals, patients, and stakeholders work together to establish and maintain standards and goals to achieve optimal health outcomes. Communication is a crucial element of healthcare delivery as it ensures that patients receive appropriate dental care, understand their diagnoses and treatment options, and feel supported throughout the care process [3]. This review aims to review the importance, challenges, strategies, and training of dentist–patient communication. Effective communication involves precise listening or observation of both verbal and non-verbal responses. This not only improves the accuracy of diagnoses but also promotes ethical clinical decisions and positive patient outcomes. It also encourages the patient’s utilization of services and boosts satisfaction levels between patients and clinicians [4]. Effective communication is pivotal in dental care as the overall success of the dental practice hinges on dentists’ ability to comprehend patients as unique individuals, perceive their wants and needs, and guide them toward informed decisions that serve their best interests. On the contrary, previous literature has provided robust evidence linking communication breakdowns between dentists and patients to malpractice claims [5]. It is noteworthy that such adverse outcomes can be mitigated through the enhancement of the dentist–patient relationship via effective communication.

## 2. Literature Search

An informal search for articles with keywords: ‘dentist-patient communication OR medical communication OR dentist–patient interaction AND patient care’ was performed on the electronic databases PubMed, Web of Science, Scopus and Google Scholar. The search was tested and refined based on the main research question and key components. A total of 72 articles that are related to dentist–patient communication in the English language were included in this overview.

## 3. Benefits of Dentist–Patient Communication

**Figure 1:**
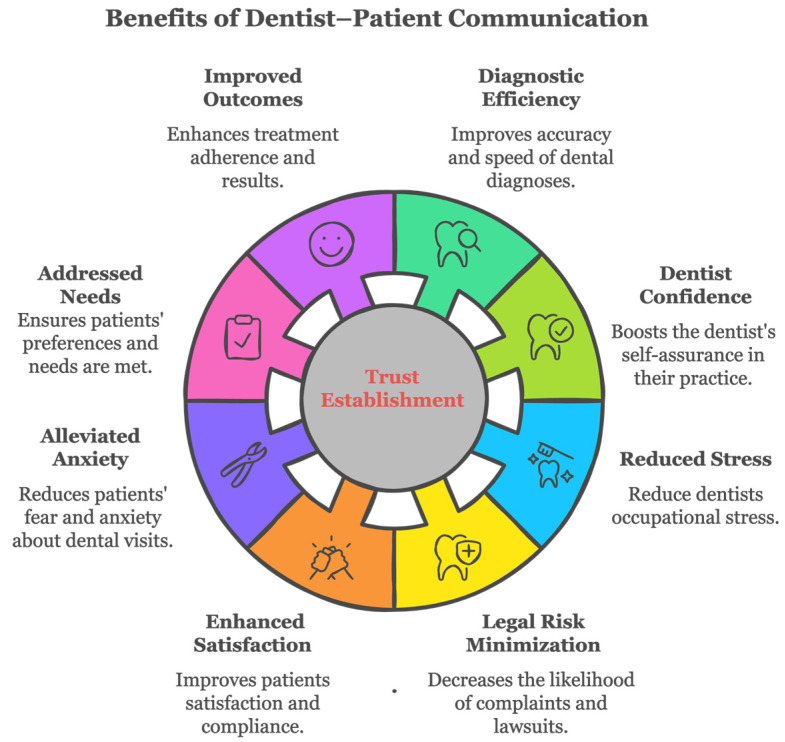
Benefits of effective dentist–patient communication in dental practice.

Good dentist–patient communication brings considerable benefits to both dentists and patients. It allows dentists to accurately and effectively pass on essential oral healthcare information to patients to enhance the quality of dental care provided to patients. It improves dentists’ efficiency, boosts self-confidence, reduces occupational stress, and minimizes the risks of complaint or litigation (Figure 1).

Moreover, it alleviates dental anxiety and fear, helps build trust between dentists and patients, addresses patients’ needs and preferences, and increases patients’ adherence to improved treatment outcomes. Effective dentist–patient communication empowers patients with the knowledge to decide the most suitable treatment and improve patient satisfaction. Most of the benefits are not clear-cut to one another but are closely intertwined.

### 3.1. Improve Diagnostic Efficiency and Accuracy

Effective communication between dentists and patients can improve the diagnostic efficiency and accuracy of a dentist. Dental professionals can make use of telecommunication applications to evaluate patients’ understanding and their acceptance level of dental advice. This enables dental professionals to gain a better understanding of patients’ concerns, preferences, and overall health status remotely and in a timely manner. A study on remote diagnoses using intraoral scans in tele-dentistry indicated that the utilization of tele-dentistry was effective in identifying dental findings and facilitated a time-efficient screening and triage process for patients [6]. Additionally, research conducted by Aboalshamat et al. demonstrated that tele-dentistry is a dependable method for accurately documenting a patient’s chief complaint and medical history and assessing the number of missing and filled teeth [7]. Effective communication between dentists and patients facilitated by tele-dentistry leads to a mutual understanding of patient expectations and treatment goals, reducing the likelihood of misunderstandings and ensuring that dental care decisions align with patients’ needs and expectations.

### 3.2. Boost Dentists’ Confidence

Good dentist–patient communication can lift confidence in dentists for handling patients’ emotions. A study on clinicians’ practice in breaking bad news of oral cancer suggested that clinicians with more experience and specialty training in communication are more able to deliver bad news with confidence and compassion [8]. Furthermore, effective communication enables dentists to have better treatment planning. When a dentist can communicate effectively with their patients, they can better understand patients’ needs, concerns, and goals. Dentists will then be able to provide more personalized and effective treatment options to their patients. This will lead to improved treatment outcomes and increased patient satisfaction [9]. In general, dentists who can achieve desirable treatment outcomes for their patients and gain high satisfaction from their patients tend to exhibit greater confidence in their practice because the positive treatment outcomes serve as justifications for their diagnostic approach and reinforce their confidence in their professional competence. Consequently, dentists have higher confidence in delivering high-quality dental care and tend to approach patients in a proactive and positive manner. They may display a greater willingness to recommend treatment plans beneficial for their patients.

### 3.3. Reduce Dentists’ Occupational Stress

Effective communication reduces occupational stress in dentists as it helps dentists stay away from the tension and anxiety at work [9]. Given that dentists usually work under busy schedules, it is inevitable for dentists to experience a certain level of occupational stress [10] when they have to perform technically complex tasks as well as manage the meetings with patients within a limited time slot. Studies indicate that communication training can play a crucial role in mitigating burnout among physicians [11,12]. Effective communication is essential for dentists in handling patient emotions and conveying sensitive dental information, particularly when discussing stigmatized medical conditions with patients [13]. Furthermore, another piece of research highlighted communication obstacles as hurdles faced by numerous physicians and suggested ongoing educational initiatives to enhance communication abilities with patients and their families, which could help alleviate job-related stress [14].

### 3.4. Minimize Risk of Complaint and Litigation Against Dentists

Dentists’ rights can be protected through the cultivation of friendly relationships with patients and the establishment of sufficient communication as these measures have the potential to reduce the rate of litigation [15]. It is also crucial for obtaining informed consent and addressing any ethical concerns that may arise during the course of care. Substantive evidence shows that malpractice complaints have no direct bearing on the technical competence of dentists [16] but are highly related to insufficient communication or communication breakdown between dentists and patients [5,17]. Another study underscored the importance of the interpersonal behaviors of dentists, claiming that their attitudes and communicative styles can also have strong connections to malpractice complaints [18].

### 3.5. Establish Trust in Dentists and Patients

Effective communication helps build trust and rapport between dentist and patient, fostering a strong therapeutic relationship [19]. Patients who are given clearer explanations and pertinent medical advice tend to build more trust in dentists [20]. Trust plays a pivotal role in dental care encounters as it is important for patients to feel comfortable sharing his or her personal information or feelings and concerns. A study on patients’ trust in physicians when assessing healthcare quality reported that patients consider trust as the main characteristic in building personal rapport and strong relationships with their dentists [21]. Moreover, patients having trusting relationships with dentists are inclined to pay more attention to their oral health and visit their dentists regularly [22]. On the other hand, the dentist needs to gather accurate information from their patients to make correct diagnoses and develop appropriate treatment plans. Good communication skills enable dentists to ask the right questions, listen attentively to the patient’s responses, and understand their illness, concerns, and needs.

### 3.6. Alleviate Dental Anxiety and Fear

Effective communication can alleviate dental fear or dental anxiety in patients. According to the vicious circle of dental anxiety, dental anxiety initially stems from the avoidance of dental visits. Patients develop feelings of inferiority and shame as their oral health deteriorates. As a result, they further postpone their treatments [23]. However, numerous studies have confirmed that dental anxiety can be dealt with by the good communication skills of dental care providers [20,24,25]. Effective communication not only facilitates information exchange but also enhances patients’ understanding of their dental situations and the corresponding treatment advice given by the dental care providers. Previous literature highlighted that the level of dental fear in patients can be significantly reduced if they have a thorough understanding of the treatment processes [20] and frequent information sharing with dentists [5].

### 3.7. Address Patients’ Needs and Preferences

Effective communication allows dentists to appreciate and respect the cultural backgrounds and beliefs of patients. This helps ensure that the dental care provided is tailored and patient-centered to meet individual needs and preferences. Similarly, effective communication enables dentists to address and manage any concerns and distress that patients may have about changes in dental care [26].

### 3.8. Improve Patients’ Adherence and Treatment Outcomes

Patients’ adherence is influenced by various factors, and patient–physician communication is one of them. Effective patient–physician communication increases patients’ adherence to medical treatment [27,28] to improve treatment outcomes. Good communication skills allow dentists to communicate complex dental health information to their patients in a way that is clear and easy to understand. Open communication allows patients to clarify their queries with physicians by encouraging them to ask questions [27]. When a patient understands his or her diagnosis and the reasons for a specific treatment plan, they are more willing to follow the dentist’s recommendations [29]. Effective communication ensures that the patient knows what to expect and how to manage his or her oral health condition effectively. Effective communication between dentists and patients yields numerous advantages for both parties. Many of these benefits, such as increased patient satisfaction [30], fostering strong patient–dentist relationships, and alleviating dental anxiety and fear in patients [31], have been shown to have a positive correlation with patient adherence to treatment recommendations.

### 3.9. Enhance Patient Satisfaction

There is ample evidence of the positive relationship between dentists’ communication skills and patient satisfaction [5,32]. Patients who feel understood, respected, and involved in their dental healthcare are more likely to feel satisfied. Good communication skills can be reflected by the amount of time the dentist spends on giving oral health information and advice to the patients. A study on patient satisfaction corroborated that the longer the time the physician spends on dental health discussions with their patients, the higher patient satisfaction they will gain [33]. Moreover, another study on patient satisfaction and compliance demonstrated that patient satisfaction is significantly affected by how patients perceive dentists’ attitudes and their communicative styles. They reported that physicians who are perceived as effective and empathic in their communication are often rated with higher satisfaction [34].

## 4. Challenges of Dentist–Patient Communication

**Figure 2:**
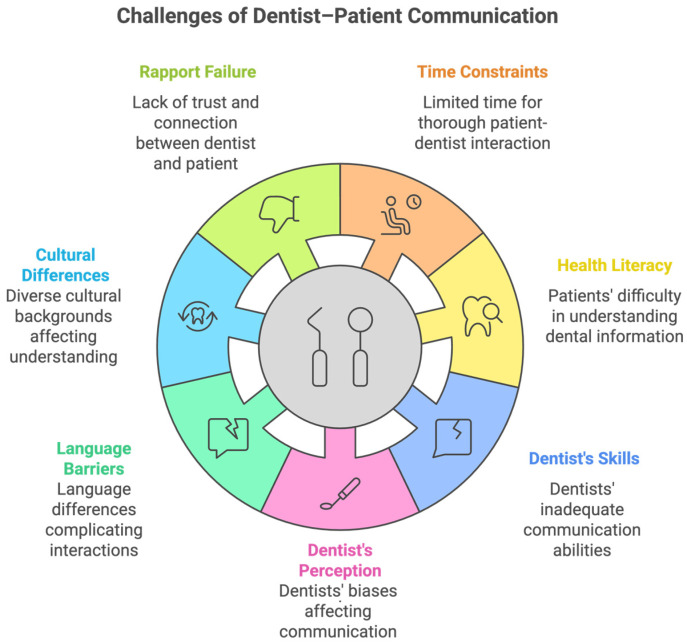
Challenges of dentist–patient communication in dental practice.

All dentists, to some extent, experience the fallout of ineffective communication. There are several factors that hinder effective dentist–patient communication. Time constraints, failure in establishing rapport, the limited health literacy of patients, the poor communication skills of dentists, dentists’ perceptions, language barriers, and unfavorable physical dental settings are common challenges for dentist–patient communication in dental practice. Figure 2 shows the challenges of dentist–patient communication in dental practice.

### 4.1. Time Constraints

Although the length of dental appointments varies, the amount of time of dental appointments is generally not sufficient for dentists and patients to interact [35,36]. Dentists face challenges in prioritizing communication during dental visits due to the necessity of performing highly skilled and technical tasks within limited timeframes. Patients may be hesitant to address important issues if they perceive the visit as rushed or if they feel there is insufficient time for meaningful communication. A study in China revealed that physicians usually shorten the time spent on patients due to heavy workloads [37]. This can undermine otherwise friendly and sufficient communication with patients.

### 4.2. Limited Health Literacy of Patients

Previous literature has recognized the positive association between oral health literacy rate and oral health status [38,39]. Patients with limited oral health literacy tend to have a reduced capacity to assimilate health information [40,41] and are less familiar with dental concepts, leading to hindering communication with dental personnel [42]. Research [43] revealed that patients with lower oral health literacy may intentionally conceal their limited understanding due to feelings of embarrassment. This implies that patients with lower oral health literacy may be less able to communicate with dentists effectively as they tend to ask fewer questions.

### 4.3. Inadequate Communication Skills of Dentists

Previous research has identified communication gaps between healthcare providers and patients [44]. These gaps are often attributed to dentists’ insufficient communication skills in effectively engaging with their patients. A study revealed that patients could recall less than half of the oral healthcare advice provided by their dentists [45], underscoring a notable communication shortfall. This lack of comprehension can be due to dentists frequently using complex dental terminology during patient interactions, which can be challenging for patients to understand [46,47]. As a result, patients may encounter difficulties in comprehending the information conveyed by dentists regarding their oral health status and treatment instructions. Additionally, information overload can impede patients’ understanding, retention of dental advice, and adherence to recommended care. Kessels [48] proposed that an excessive amount of health information presented by practitioners could detrimentally affect patients’ memory retention of medical information.

### 4.4. Dentists’ Perceptions

Previous research Studies found that dentists consciously withhold information from their patients if they think that their patients cannot understand complex information in the form it is usually presented, if they observe that their patients are indifferent to the topic, or if they believe they should be the one to make healthcare decisions for their patients [49,50,51]. Furthermore, dentists commonly have a shared perception, wherein they tend to make assumptions about their patients’ desires rather than actively seeking to understand their patients’ specific preferences and needs [49]. This prevailing perception among dentists can lead to dental consultations commencing with unrealistic expectations on the part of patients.

### 4.5. Language Barriers

With increasing globalization, communities become more ethnically diverse. Transculturalism and language barriers thus imperil a large population worldwide in their access to quality dental care [52]. Language barriers have been identified to lengthen the time for communication and decrease empathy and approachability [53]. In addition, the inability to communicate can be a distressing and fearful experience for ethnic minority patients that leads them to avoid dental visits. Moreover, disagreements or conflicts may arise due to misunderstanding and miscommunication, which poses greater challenges to dental professionals. In addition, patients with hearing and speech impairment may refrain from interacting with healthcare providers due to communication challenges [54]. In addition to written information, some individuals rely on lip reading, finger spelling, and cue speech to communicate with healthcare providers. However, these communication methods may not be familiar to all providers, who may lack training in their use [55].

### 4.6. Cultural Difference

Cultural differences can affect communication and understanding between patient and dentist. When communication takes place between people of different races and religions, the cultural disparity can have negative impacts on effective dentist–patient communication. Cultural differences in body language can increase the risk of miscommunication between dentist and patient, given that various cultures may place distinct emphasis on particular postures, gestures, and signs [52]. What may be acceptable or normal in one culture may be offensive in another. Dental professionals need to be culturally competent when dealing with patients of different races.

### 4.7. Failure in Establishing Rapport

It is essential for dentists to establish good rapport with their patients to provide quality care. The barriers discussed above, such as language barriers [56,57], cultural barriers [58], and time constraints [58], are common hindrances in establishing rapport in patient care. In addition, dentists may have personal biases and attitudes that can affect their interactions with patients. These biases can be based on race, gender, sexual orientation, or other factors. On the other hand, patients may be hesitant to trust healthcare providers due to past negative experiences. Dentists need to build trust with patients to establish rapport [59]. Furthermore, the patient may also be dealing with emotional issues that can worsen an otherwise harmonious relationship with the dentist. Healthcare providers need to be empathetic and understanding of the patient’s emotional state.

## 5. Strategies of Dentist–Patient Communication

Enhancing communication between dentists and patients is crucial for effective dental care. Dentists should adopt a patient-centered approach as the foundation of their interactions [60]. This approach involves a deep understanding of the patient’s condition, engaging in shared decision-making, and providing interventions that respect the patient’s pace and preferences [61]. This involves not only diagnosing the disease but also explaining preventive dentistry and promoting dental health. Patients are invited to share their personal experiences with illness. In a patient-centered approach, decision-making is a collaborative effort between the dentist and the patient. Patients are encouraged to actively engage in discussions, evaluations, and validations, and to participate in co-creating their treatment plans. Dentists should provide decision support and share dental health information to build a strong therapeutic alliance [62]. To improve communication, dentists should employ simple and clear language, maintain appropriate body posture, use gestures and facial expressions, and make eye contact during interactions with patients [60]. Demonstrating empathy, encouraging questions and feedback, utilizing visual aids, and allowing sufficient time for patients to express their concerns are also essential components of effective communication in dental care. In today’s digital age, dentists and patients are increasingly utilizing messaging applications for communication. This form of telecommunication offers convenience and cost-effectiveness, reducing the need for in-person visits to the dentist’s office.

## 6. Communication Training in the Dental Curriculum

The importance of training dental students in clinical communication skills and behavioral aspects of treatment is widely acknowledged in dental education literature, primarily due to its significant clinical relevance. Incorporating communication skills training into the dental curriculum is deemed essential and is highly valued by dentists, dental students, and patients alike [5,63]. Numerous institutions provide training sessions specifically designed to enhance communication skills. However, these sessions are often primarily designed for medical students. When dental students are included in such training, there is typically no provision for assessing the learning outcomes of the training [64,65]. Dental communication curricula primarily consisted of singular didactic coursework sessions, lacking significant practical engagement for students, until notable modifications were implemented approximately a decade ago [66]. Carey and colleagues [66] also pointed out that the communication skills training in the dental curricula was often a one-time occurrence, thus limiting the students’ opportunities for progressive development of these skills. Since the knowledge acquired during the initial years of students’ academic pursuits forms the core foundation for their subsequent clinical learning experiences, it is recommended that implementing longitudinal communication strategies in dental education allows for a more comprehensive assessment of students’ communication skills throughout their study [64,67].

An optimal framework for communication skills training should encompass a structured pedagogical approach, integrated seamlessly across the entirety of the dental curriculum, incorporating a blend of didactic instruction, role-playing exercises, and practical patient interviewing as well as continuing assessments conducted by students themselves, their peers, and instructors. Various kinds of patient communication techniques have to be included in the communication skills teaching, such as generic skills, including patient-centered care, active listening, empathy, etc.; case-specific skills, including motivational medical interviewing, information sharing, etc.; time-specific skills, including session initiation, session closure, etc.; and emerging skills, including cultural sensitivity, breaking bad news, etc. [67]. The didactic integration of structured communication training within the curriculum can significantly improve students’ skills in engaging with patients effectively. An important component is the role-playing exercises. Role-playing exercises were found to be beneficial in differentiating between effective and ineffective communication skills. Studies showed that dental students display positive attitudes towards actively learning communication skills through these interactive exercises [68,69]. Another crucial component of the communication skills training in the dental curriculum is motivational interviewing with patients. Motivational interviewing plays a vital role in enhancing patient concordance and compliance [70]. Encouraging discussions about change fosters improved cooperation with patients by shifting away from simply providing instructions and advice. This approach facilitates more meaningful conversations that lead to better patient engagement and adherence to treatment plans [69]. Scholars outlined motivational interviewing as including the following four elements [71,72]:

- Open-ended questioning—Differing from the closed questions, open-ended questions enable patients to reflect and give more detailed, comprehensive responses. Initiating conversations with phrases such as “how,” “what,” or “describe” encourages patients to take the lead in the dialog.
- Affirmations—Genuine affirmations play a pivotal role in fostering a stronger rapport with patients. These affirmations, conveyed through both verbal statements and non-verbal cues, assist patients in recognizing their strengths and acknowledging behaviors that contribute to positive changes, irrespective of their magnitude.
- Reflective listening—Similarly to active listening, reflective listening signifies that the dentist has accurately comprehended and acknowledged the patient’s communication. This empathetic approach nurtures a deeper exploration of issues and emotions, promoting discussions around potential changes.

Summaries serve to reinforce the content discussed. Summarizing involves capturing a comprehensive understanding of a patient’s behaviors and verifying with the patient to ensure that the healthcare professional has accurately reflected their circumstances. This summarization process aids in consolidating the communication exchange between the patient and provider.

The incorporation of communication skills training within the dental curriculum, particularly through a longitudinal integrated teaching approach, has demonstrated significant benefits in enhancing the communication proficiency of dental students. This comprehensive approach not only equips students with essential communication skills but also fosters a patient-centered mindset essential for effective dental practice. Table 1 presents a summary of the crucial components of communication training in dental education.

## 7. Conclusions

Dentist–patient communication is at the core and essence of providing quality dental care. Effective dentist–patient communication is vital for the success of a dental practice. It is evident that dentists face challenges due to suboptimal communication strategies such as time constraints, rapport establishment difficulties, and language barriers. To address these hurdles, adopting a patient-centered approach and honing both verbal and non-verbal communication skills are essential. Utilizing simple language, maintaining positive body language, and engaging in structured communication training are key strategies to improve dentist–patient interactions and enhance patient engagement and adherence to treatment plans in dental education.

## Figures and Tables

**Table 1:** The challenges of dentist–patient communication in dental practice.

Teaching Components	Sub-Categories	Description
Didactic Instruction		Structured lectures and coursework designed to provide theoretical knowledge on communication skills.
Interactive Learning	Role-Playing Exercises	Interactive activities where students practice communication skills in simulated scenarios.
	Practical Patient Interviewing	Real-life practice through interviewing actual patients in a clinical setting.
Assessment Methods	Continuing Assessments	Ongoing evaluations are conducted by students themselves, their peers, and instructors.
Skill Categories	Generic Communication Skills	Basic communication skills applicable to a variety of scenarios.
	Case-Specific Communication Skills	Specialized skills for specific scenarios, including motivational interviewing and information sharing.
	Time-Specific Communication Skills	Skills pertinent to different stages of patient interaction, such as session initiation and closure.
	Emerging Communication Skills	Advanced skills like cultural sensitivity and breaking bad news.
Teaching Strategy	Longitudinal Integrated Teaching Approach	Continuous and comprehensive communication skills training integrated throughout the dental curriculum to ensure progressive skill development.
